# Jiang Zhi Granule protects immunological barrier of intestinal mucosa in rats with non-alcoholic steatohepatitis

**DOI:** 10.1080/13880209.2021.1979594

**Published:** 2021-12-16

**Authors:** Xiao Yu, Haiyan Zhang, Jielu Pan, Lu Zou, Ling Tang, Hongyu Miao, Peiyong Zheng, Lianjun Xing

**Affiliations:** a Department II of Digestive Diseases, Longhua Hospital, Shanghai University of Traditional Chinese Medicine, Shanghai, China; b Experiment Center for Teaching & Learning, Shanghai University of Traditional Chinese Medicine, Shanghai, China; c Institute of Digestive Diseases, Shanghai University of Traditional Chinese Medicine, Shanghai, China

**Keywords:** MLNs, DSS, TLR4–MyD88

## Abstract

**Context:**

Jiang Zhi Granule (JZG) is known to improve hepatic function, reduce liver fat deposition and inflammation in non-alcoholic fatty liver disease (NAFLD).

**Objective:**

To determine the protective mechanism of JZG on immunological barrier of intestinal mucosa in rats with diet-induced non-alcoholic steatohepatitis (NASH).

**Materials and methods:**

A Sprague-Dawley (SD) model of NASH was established using a high-fat diet and 1% dextran sulphate sodium (DSS) through drinking water. The rats were randomized into four groups and treated for four weeks, respectively, including normal control (NC), model control (MC), positive control (PC) and JZG. Mesenteric lymph nodes (MLNs) cells were isolated and cultured to assess a potential disruption of the enteric immune barrier. Also, investigation of intestinal mucosal dendritic cell-toll-like-receptor-myeloid differentiation primary response 88 (DC-TLR-MyD88) signalling pathway *in vitro* was examined.

**Results:**

The lethal concentration 50 (LD_50_) of JZG was greater than 5 g/kg, while its inhibitory concentration 50 (IC_50_) was 1359 μg/mL in HepG2. In JZG group, the plasma levels of alanine transaminase (ALT), aspartate transaminase (AST), malondialdehyde (MDA), low-density lipoprotein cholesterol (LDL-C), total cholesterol (TC), triglyceride (TG) and serum endotoxin were significantly (*p* < 0.01) reduced. In contrast, plasma concentrations of high**-**density lipoprotein cholesterol (HDL-C) and superoxide dismutase (SOD) were increased. Furthermore, proinflammatory factor, interferon-γ (IFN-γ)+ from CD4+ T cells in DSS-induced NASH rats increased significantly (*p* < 0.01) compared to NC group. Importantly, JZG treatment substantially decreased (*p* < 0.01) the relative expressions of TLR-44 and MyD88.

**Conclusions:**

JZG treatment may protect immunological barrier of intestinal mucosa in NASH individual.

## Introduction

A clinical syndrome with the umbrella term non-alcoholic fatty liver disease (NAFLD) has been characterized via hepatic steatosis of parenchyma and storage of fat without any history of excessive drinking of alcohol (Patel et al. 2011). It encompasses a spectrum of diseases with its pathogenesis mainly associated with insulin resistance (IR) and metabolic syndrome (MS) (Alkhouri et al. 2010). The incidence of liver steatosis has been reported to increase annually probably because of changes in living standards of individual. Clinically, NAFLD progresses from simple steatosis to non-alcoholic steatohepatitis (NASH), which culminates in liver cirrhosis and even hepatocellular carcinoma (Lombardi et al. 2017). The main risk factors for the development of NASH are obesity, hypertension, hypertriglyceridaemia and IR (Lo et al. 2015; Younossi et al. 2018). Usually, patients with NASH often develop fibrosis and cirrhosis with its pathogenesis being extremely complex. The theory of ‘two strikes’ put forward by Day (Wang et al. 2015) has become the main hypothesis to elucidate the pathogenesis of the disease. Based on various reasons, the first hypothesis suggested that increased accumulation of lipids in liver culminated in the occurrence of simple steatosis. The second theory indicates that an increase in the generation of free radicals and lipid peroxidation could result in the overproduction of some cytokines, which may directly damage hepatocytes and cause inflammation and necrosis.

Jiang Zhi Granule (JZG) is a clinically prescribed traditional Chinese Medicine for the treatment of patients with NAFLD. Jiang Zhi Granule is composed of *Salvia miltiorrhiza* Bunge (Lamiaceae) (24 g), *Folium nelumbinis* (6 g), *Polygala tenuifolia* Willd. (Polygalaceae) (60 g) (Song et al. 2013), *Artemisia capillaris* Thunb. (Asteraceae) (6 g) and *Gynostemma pentaphyllum* (Thunb.) Makino (Cucurbitaceae) (60 g) (Song et al. 2013). Drug safety evaluation of JZG has shown positive result, while its clinical trials have been approved by the State Food and Drug Administration (SFDA, the authorized number: Z10960082). Previous studies have established that JZG has a definite effect on the improvement of fat accumulation in the cell lines and liver of animals (Zheng et al. 2018a, 2018b).

Lipid accumulation in liver is one of the markers of NASH (Chavez-Tapia et al. 2012). The initial step is due to excessive transport of free fatty acids to adipose tissue coupled with the imbalance between lipid synthesis and export to hepatocytes, which consequently culminate in the accumulation of fat in the liver. However, the role of fat accumulation as an integral part of influencing and sensitizing liver to further injury has not been completely clarified. Endotoxin or lipopolysaccharide (LPS) is a bacterial wall component sensed by toll-like receptor-4 (TLR-4) (Csak et al. 2011; Seeley and Ghosh 2017) and has the capacity to cause progressive liver injury. Endotoxin tolerance is generally decreased in response to the LPS challenge after first exposure to the toxin which capably prevent fatal LPS challenge, infection and ischemia–reperfusion injury. Generally, it is accepted that the innate cells of the immune system, namely macrophages and dendritic cells (DC), highly express LPS which recognizes TLR-4, but lose their ability to secrete high levels of pro-inflammatory cytokines after exposure to endotoxin-tolerant LPS. Indeed, TLR is an important receptor family (Ji et al. 2018) that enables the innate immune system to respond immediately to infection by identifying bacterial and viral components. In addition, the importance of bacterial DNA receptors such as TLR-4 has been confirmed in NASH (Allard et al. 2009). Moreover, the complexity of the role of gut microflora in the maintenance of human health and disease incidence has indicated that NAFLD is a distinctive enteric microflora-related disease. Thus, the gut-liver axis theory has suggested the linkage between intestinal tract and hepatic diseases. The gut mucosal immune system must usually maintain the balance between harmless responses to food and symbiotic bacteria as well as protective immunity against invasive pathogens. This system serves as a protective barrier for the intestinal tract integrity and comprises epithelial cells, lamina propria and lymph nodes (Shi et al. 2017). In recent times, researchers investigated the possible role of immunological barrier dysfunction of the intestinal mucosa in NAFLD pathogenesis and have suggested the protection of the intestinal barrier function as a novel approach to treat NAFLD and its associated conditions (Cui et al. 2019). In particular, available evidence (Thome et al. 2017) has proven that mesenteric lymph nodes (MLNs) resident immune cells play important roles in peripheral immune tolerance in the gut. Thus, DC is believed to act as a bridge between innate and acquired immunity, which is at the centre of initiation and regulation of specific immune responses (Ma et al. 2013). Also, DC is reported to determine types of immune responses, such as inducement of Th 1, Th 2, Th 17 and Treg production (Turner et al. 2014; Azevedo et al. 2017). Most of the DCs have been found to be immature, while TLRs expressed on the surface of DCs are stimulated by antigen ligands with intracellular transmission of information firstly to MyD88 protein (Yao et al. 2012; Mbongue et al. 2017). The activation of DC coupled with the activated MyD88 phosphorylates the downstream related proteins, induces maturation of DCs, and promotes the secretion of co-stimulatory molecules as well as other related cytokines, which in turn activates T cells and induces specific immune response (Pan et al. 2017). Importantly, TLR4–MyD88-mediated the initiation of nuclear factor-κB (NF-κB) activation and concomitantly triggered host response to infection-induced inflammation via proinflammatory genes (Jack et al. 2005; Zughaier et al. 2005).

Based on the aforementioned literature, we suggested that JZG may protect immunological barrier of intestinal mucosa in rats with NASH. In this regard, we monitored changes in DC levels after induction of NASH, while the ability of naïve CD4+ T cells to differentiate into CD4+ T helper cell subsets (Th 1, Th2) was also investigated. Herein, it was postulated that enteric DC could induce damage in the intestinal mucosal immune barrier in NASH. Also, through the regulation of TLR4–MyD88 signalling pathway, damage to intestinal mucosal immune barrier by NASH may be prevented via JZG treatment.

## Materials and methods

### LD_50_ and IC_50_

Rats were randomly divided into blank and JZG groups with the latter further categorized into 0.5, 1, 5 and 10 g/kg doses, wherein each group comprised of eight rats (four females and four males). After fasting for 10 h, the rats in the JZG group intragastrically received 0.5, 1, 5 and 10 g/kg of JZG, while those in the blank group were given equal volume of distilled water via the same route. The intragastric volume of different groups was given at 0.1 mL per 10 g body weight, prior to fasting of the rats for 2 h after administration, and observation for seven days. Notably, no death was observed among the rats when the dose was higher than 5 g/kg by body weight, indicating that the LD50 of JZG was greater than 5 g/kg.

**Figure 1.**
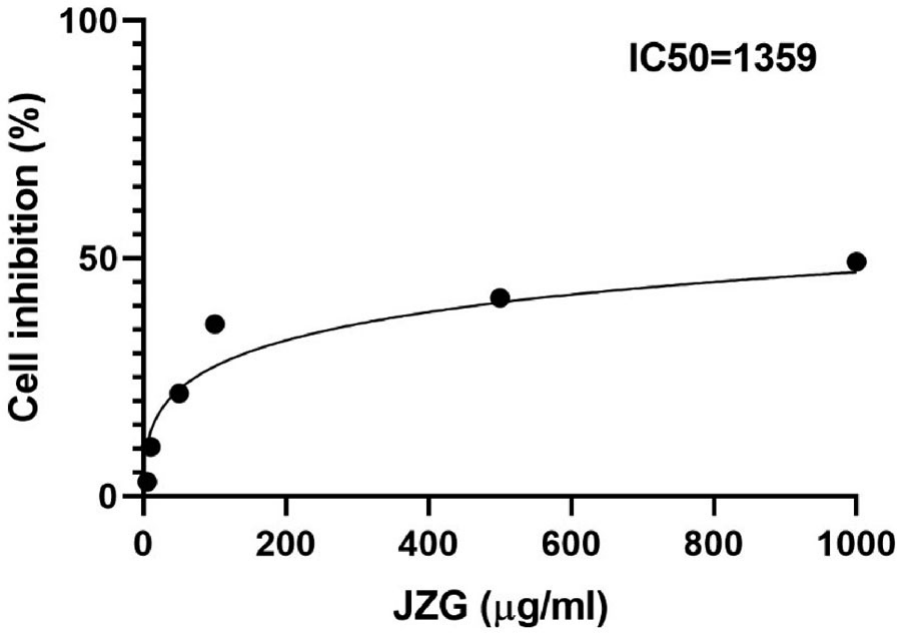
Half maximal inhibitory concentration (IC_50_) of JZG in HepG2.

To evaluate the cell bioavailability potential of the medicine, we treated the HeG2 with JZG. HepG2 was seeded at 2 × 10^5^ cells per mL in the 96 well for 100 µL per well. When the confluence attained 90%, different concentrations of JZG (0.5, 1.0, 2.0, 5.0, 10, 50 and 100 µg/mL) were prepared with the medium as solvent before addition to each well. MTT was applied to count the inhibitory activity of JZG, thus inhibitory concentration 50 (IC_50_) was calculated with GraphPad Prism 8.0 (La Jolla, CA). As shown in Figure 1, IC_50_ of JZG was 1359 µg/mL in HepG2.

### Establishment of a high-fat dietary dextran sulphate sodium (DSS) steatohepatitis model

Eighty male Sprague-Dawley (SD) rats (150 ± 10 g) from the Laboratory Animal Centre of Jiangsu University (Zhenjiang, China), were randomly divided into four groups with 20 rats in each group to exclude individual differences. The animals were raised in separate cages at temperature ranging 22–28 °C as described in previous literature. The four groups were normal control (NC), model control (MC), positive control (PC) and JZG. With the exception of the first group, all the rats in the other groups received high-fat diet and 1% DSS through their drinking water. However, rats in the NC were fed with normal diet. The composition of high-fat diet was as follows: 1.5% cholesterol, 0.25% sodium cholate, 10% lard, 5% sucrose and 83.25% basic feed (Jiangsu Synergetic Pharmaceutical Bioengineering Co., Ltd., Nanjing, China). The DSS was administered periodically with each cycle consisting of seven days of DSS administration followed by a 10 days interval with unrestricted access to normal drinking water. These cycles were repeated throughout the 12 weeks experimental period (Gabele et al. 2011). The animal experiments in this study were approved **(**protocol number: PZSHUTUK210115017) by the Institutional Animal Care and Use Committees of Shanghai University of Traditional Chinese Medicine.

After modelling, probiotics (*Lactococcus lactis* jcm5805, 100 mg/kg/d) were given to rats in the PC group, while those in the MC and JZG groups respectively received the same amount of normal saline, and JZG (0.828 g/kg) after dissolution in normal saline via oral gavage (Wang et al. 2013). Four weeks after administration, the rats were anaesthetized prior to the harvesting of organs and collection of blood samples from the portal vein. Tissue samples were then fixed in 10% formalin or preserved in liquid nitrogen and stored at −80 °C.

### Isolation and culture of mesenteric lymph nodes cells

All the MLNs were obtained from the model rats. Next, the MLNs were cut into small pieces and added to digestive juice. The digestive juice comprising PBS and 2% foetal bovine serum (FBS) was dissolved in 2 mg/mL type II collagenase and 50 µg/mL type I DNA enzyme. Digestion of the cells was performed at 37 °C for 45 min. Then, the cells were filtered through a cell strainer (100 µm). The target cells were collected using the anti-OX62 magnetic beads and MS + positive column (Krysko et al. 2004; Chen et al. 2016). The collected cells were observed with transmission electron microscopy (TEM) and scanning electron microscopy (SEM).

### Histopathology examination of hepatic and intestinal tissues

During the histological examination procedure, liver and colon tissues were fixed in 10% formalin, dehydrated, paraffin-embedded and cut into 4 µm thick section. The sections were stained with haematoxylin and eosin (H&E) and observed via optical microscope.

### Biochemical assays

The plasma levels of alanine transaminase (ALT), aspartate transaminase (AST), malondialdehyde (MDA), high-density lipoprotein cholesterol (HDL-C), low-density lipoprotein cholesterol (LDL-C), superoxide dismutase (SOD) inhibition rate, serum endotoxin, total cholesterol (TC) and triglyceride (TG) were determined using Elisa kit based on the specifications of the manufacturer (Nanjing JianCheng Institute of Biotechnology, Nanjing, China).

### Flow cytometry

Flow antibody labelling of MLN-DCs surface markers, viz., CD80+, CD86+ and MCH-II+ % was performed via flow pattern analysis using Beckman Coulter PC 500 MPL flow meter.

Through cell differentiation test, the CD4+ T cells from healthy untreated rats MLNs were obtained. The co-stimulatory medium was composed of RPMI1640 with 10% FBS, glutamine and double antibodies (penicillin and streptomycin). Under the condition of 5% CO_2_ and 37 °C, the CD4+ T cells (1 × 10^6^) were co-stimulated with OX62 + MLN-DCs (1 × 10^5^) (DCs from MLNs) and SPDCs (1 × 10^5^) (DCs from spleen).

Next, the CD4+ T cells were co-stimulated with phorbol 12-myristate 13-acetate and ionomycin for 4 h. Foxp3 staining kit was applied to detect the number of CD4+ T cells (representing Th1 cells and Th2 cells), while the production of cytokines (IFN-γ and IL-4) was evaluated with flow cytometry. Homologous control antibodies were used to label the background fluorescence.

### Detection of RNA expression by real-time polymerase chain reaction (RT-PCR)

The conventional primers were obtained from Shanghai Xiyuan Biological Technology Co., Ltd. (Shanghai, China), while other reagents were supplied by Shanghai General Biotech Co., Ltd. (Shanghai, China). Reverse transcriptase reaction and RT-PCR were carried out according to the manufacturer’s instruction. The primers used in this work are shown in Table 1. Reaction conditions of PCR were 95 °C (10 min), 95 °C (15 s), 60 °C (1 min) and 72 °C (1 min) at 40 cycles.

**Table 1.** Primer sequences.

Name	Forward/reverse	Primer sequence (5′ to 3′)
TLR-4	F	TGGTTCACTGGAACACCAAA
	R	AGCAAGGGTCGAAGTTAGCA
MyD88	F	TGGCCTTGTTAGACCGTGA
	R	AAGTATTTCTGGCAGTCCTCCTC

MyD88: myeloid differentiation primary response 88; TLR-4: toll-like receptor 4.

### Statistical analysis

The data were analysed with SPSS 22.0 software (Chicago, IL) and expressed as means ± standard deviation. GraphPad Prism 8.0 (La Jolla, CA) was used to draw the graphs. Comparison between multiple samples was analysed with one-way ANOVA. A *p* < 0.05 was considered to indicate a statistically significant level.

## Results

### Establishment of NASH model by DSS

**Figure 2.**
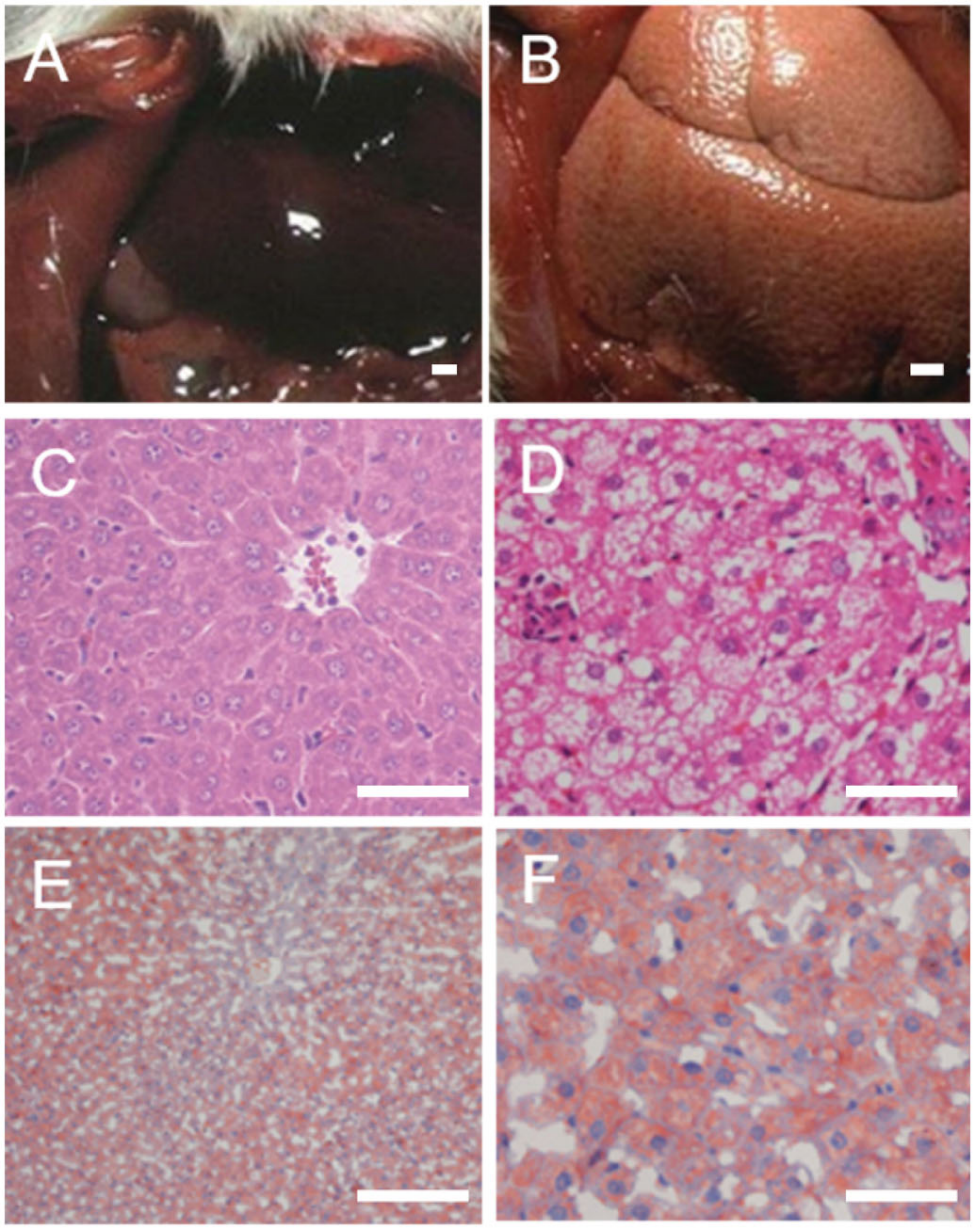
Morphological and pathological detection of liver tissue in rats (A: normal control (NC); B: model control (MC); C: haematoxylin and eosin (H&E) staining of NC; D: H&E staining of MC; E: oil red staining of NC; F: oil red staining of MC).

It was observed that the rats in the NC group had ruddy liver colour with the edge of the peripheral organs being clear (Figure 2(A)). In the MC group, the liver was yellow in colour, enlarged with nodules and adhesion, with the surrounding tissues being observed on the hepatic surface (Figure 2(B)). Paraffin sections of the rat liver tissue stained with H&E were observed in the NC group, which revealed uniform colour of the liver, while the hepatocyte cord was regularly arranged with hepatic nodules (Figure 2(C)). In contrast, large areas of fatty degeneration on liver tissue, severe vacuolation of hepatocytes around central vein and severe inflammatory reaction in localized area were observed in the liver of the rats in the MC group (Figure 2(D)). Based on the oil red O staining technique, no staining of liver tissue was observed in NC group (Figure 2(E)) while a large number of lipid droplets were distributed widely in the liver tissue of rats in MC group (Figure 2(F)). Taken together, these results indicated that the NASH model was established successfully.

### Histopathology examination of hepatic and intestinal tissues

**Figure 3.**
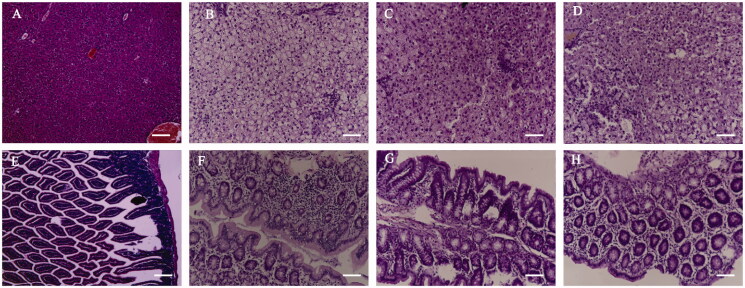
Haematoxylin and eosin (H&E) staining of liver and colon sections (A: liver in normal control (NC); B: liver in model control (MC); C: liver in positive control (PC); D: liver in Jiang-Zhi granule (JZG); E: colon in NC; F: colon in MC; G: colon in PC; H: colon in JZG).

The H&E staining of rat liver and colon is shown in Figure 3. In the NC group (Figure 3(A)), the hepatic tissue structure was intact with liver cord neatness, while the hepatic lobule structure was regular with clear boundary, and no deposition of lipid droplets in the cells. In the MC group (Figure 3(B)), the fatty degeneration of liver tissue was obvious, with disturbance of hepatic cord, infiltration of inflammatory cells in hepatic lobules and portal areas, as well as hepatocyte swelling and different sizes of lipid droplets in the cytoplasm. The main type of lipid droplets was bullous fat droplets, while the nucleus was pushed to the edge. It was difficult to identify the normal hepatocytes. The aforementioned changes indicated that NASH model was successfully established demonstrating moderate to severe steatosis and pathological alterations. The lipid droplet vacuoles in liver tissue of rats in the PC and JZG groups were reduced in varying degrees with the same degree of improvement in the infiltration of inflammatory cells in hepatic lobule and portal area in comparison with the model group (Figure 3(C,D)).

In the NC group, the mucosal layer of the colon tissue was intact with straight tubular glands on the surface of the mucosa, while no inflammatory cell infiltration was observed (Figure 3(E)). In MC group (Figure 3(F)), a large number of neutrophils and lymphocytes infiltrated the mucosa and sub mucosa, while granulation tissue was formed by collagen fibres and fibroblasts with some epithelial tissue exfoliated and necropsied. The inflammatory cell infiltration in PC and JZG groups was better than that in the MC group with more intact mucosal tissue (Figure 3(G,H)).

### Biochemical assays

**Figure 4.**
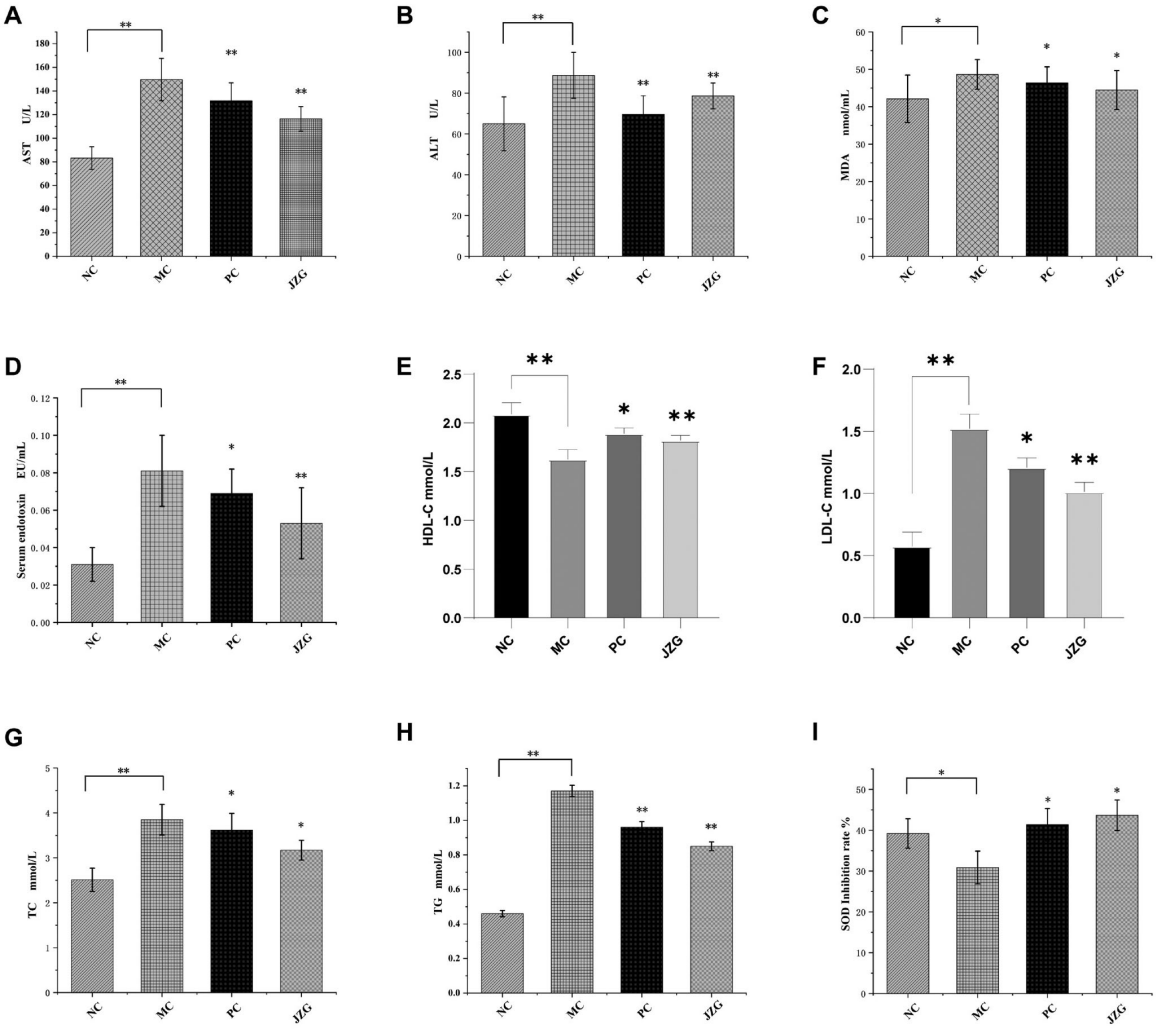
Detection of serum indicators in rats [(compared with model control (MC), *p < 0.05, **p < 0.01), normal control (NC), model control (MC), positive control (PC) and Jiang-Zhi Granule (JZG)].

The plasma levels (of AST, ALT, MDA, HDL-C, LDL-C, TC and TG) and serum endotoxin as well as reduction of SOD content were measured in each of the rats in the various groups and the results displayed in Figure 4. Compared with NC, the levels of AST, ALT, MDA, serum endotoxin, LDL-C, TC and TG in the MC group increased significantly, while HDL-C and SOD levels decreased substantially. Collectively, these results suggest successful establishment of the model in SD rats. Compared with the MC, the JZG supplementation could substantially (*p< 0.05, **p< 0.01) reduce the levels of ALT, AST, MDA, serum endotoxin, LDL-C, TC and TG, while concentrations of HDL-C and SOD were increased. The results imply that JZG had a better therapeutic effect on DSS-induced NASH in rats through effective reduction of the levels of serum ALT, AST, serum endotoxin, LDL-C, TC and TG in NASH rats, as well as increased serum HDL-C and SOD levels. Overall, these findings may promote free radical scavenging, reduction of the level of oxidative stress product (MDA), and decrease direct damage of hepatocytes by MDA.

### Identification of maturity and function of MLN-DCs in rats

**Figure 5.**
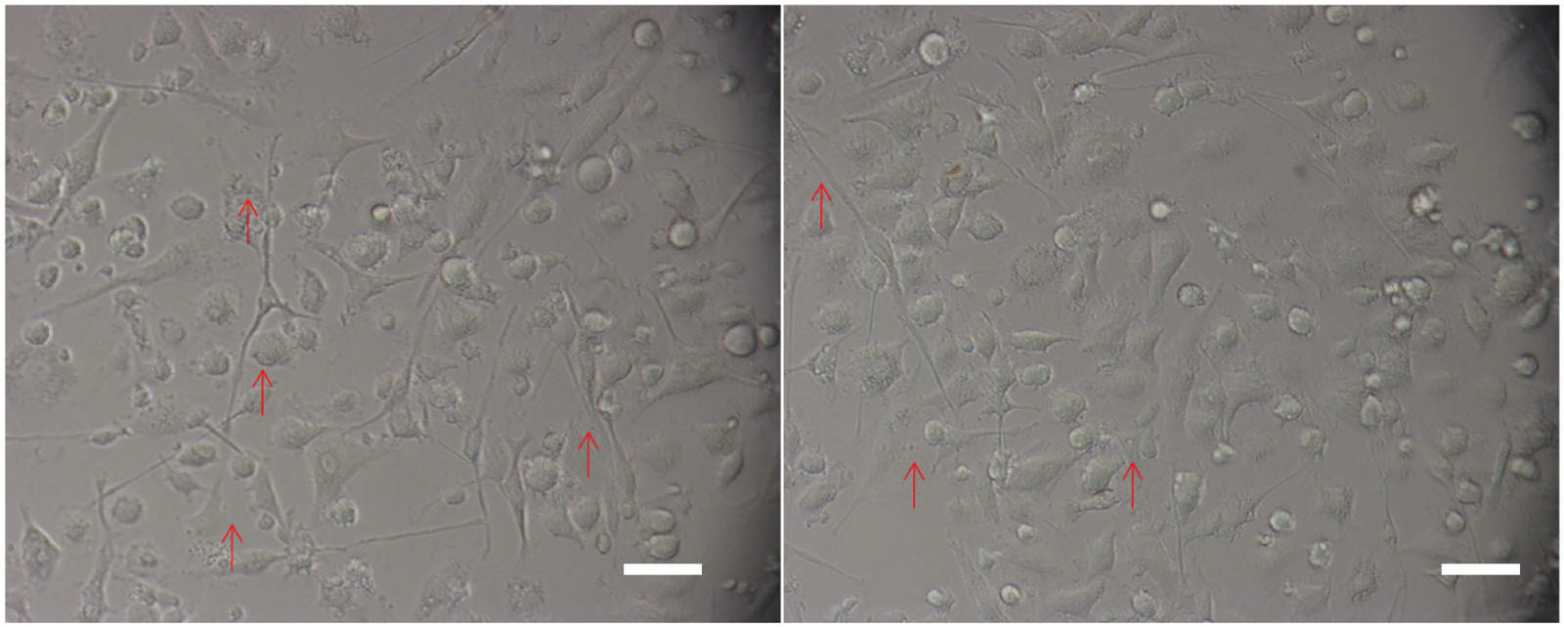
Morphology of mesenteric lymph nodes-dendritic cells (MLN-DCs).

**Figure 6.**
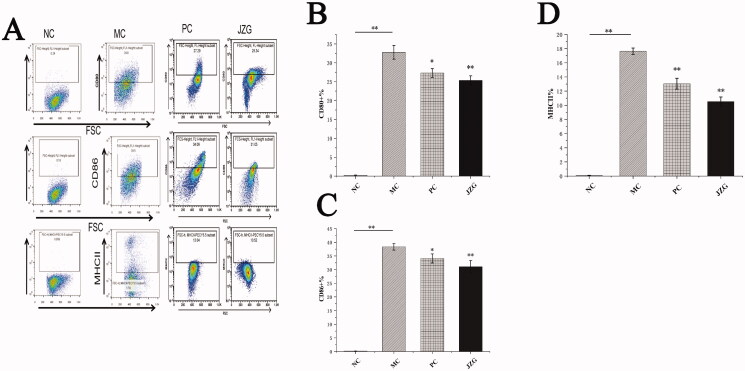
Flow cytometric detection and value of CD80+, CD86+ and major histocompatibility complex II positive (MHC-II+) in rats of each group [(compared with model control (MC), **p < 0.01, *p < 0.05), normal control (NC), MC, positive control (PC), Jiang-Zhi Granule (JZG)].

The observation of MLN-DCs morphology is shown in Figure 5. Mature DC cells were irregular and have dendritic or pseudopodal processes. To further explore the maturation of MLN-DCs, the expressions of CD80+, CD86+ and MCH-II + were analysed via flow cytometry (Figure 6). The expression of CD80+ in MLN-DCs was 0.23%±0.04 (NC group), 32.77%±1.80 (MC group), 27.49%±1.15 (PC group) and 25.34%±1.20 (JZG group). Statistically, CD80+ expression in the MC group was significantly higher than the NC (*p* < 0.01), PC (*p* < 0.05) and JZG groups (*p* < 0.01).

The expression of CD86+ in MLN-DCs was 0.19%±0.01% (NC group), 38.33%±1.20% (MC group), 34.03%±1.65% (PC group) and 31.05%±2.29% (JZG group) with statistical results indicating substantial higher expression in the MC group than that of NC (*p* < 0.01), PC (*p* < 0.05) and JZG groups (*p* < 0.01).

In addition, the expression of MCH-II in MLN-DCs was 0.07%±0.05 (NC group), 17.63%±0.45 (MC group), 13.04%±0.77 (PC group) and 10.52%±0.65 (JZG group). This result showed that the expression of MCH-II + in MLN-DCs (MC, PC and JZG groups) was significantly higher compared with the NC group (*p* < 0.01).

### Number of CD4+ T cells releasing IFN-γ+ and interleukin 4 (IL-4)+ after co-stimulation

**Figure 7.**
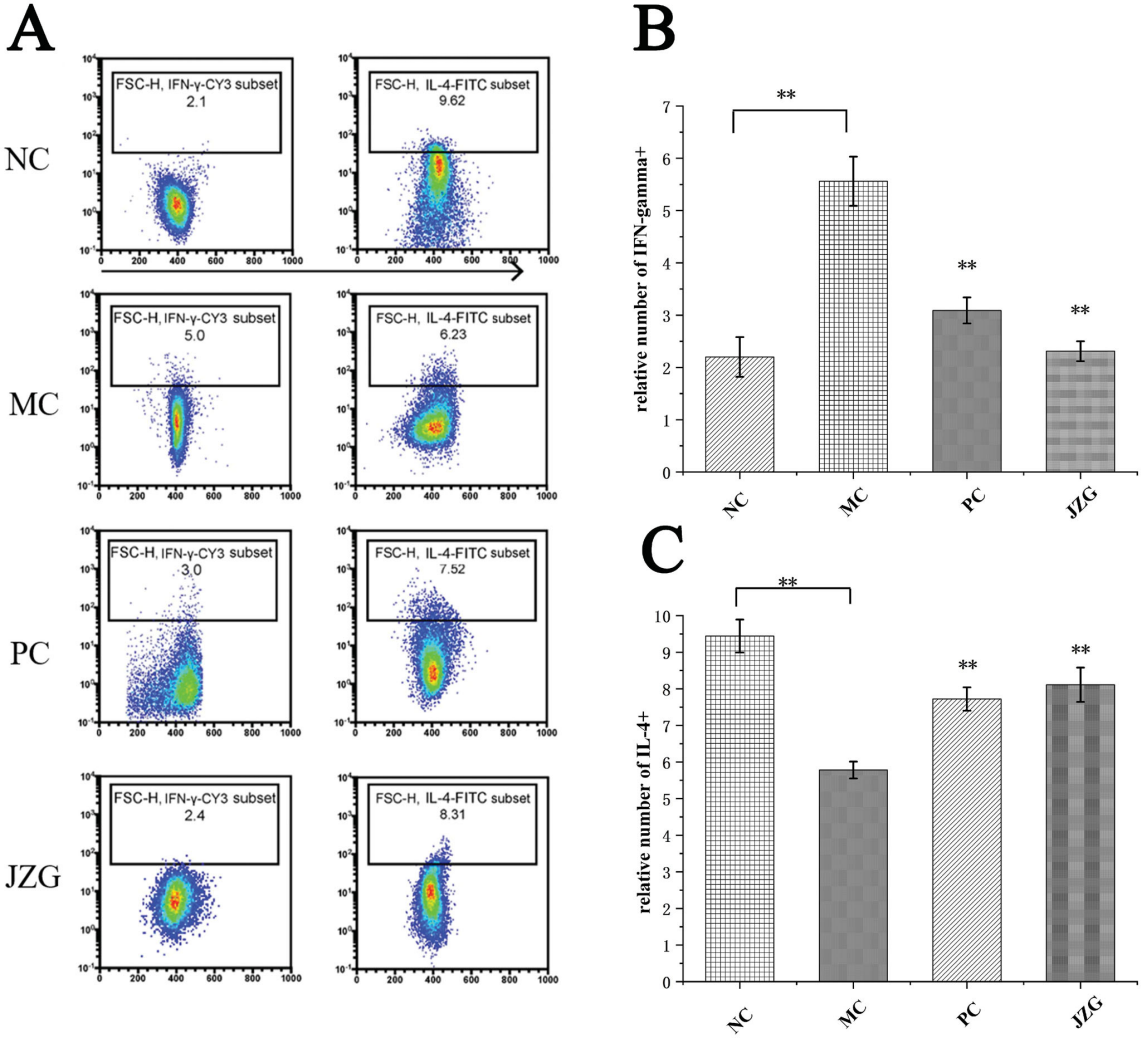
Flow cytometry was used to detect the number of CD4+ T cells co-cultured with interferon gamma positive (IFN-γ+) and interleukin 4 positive (IL-4+) cells (compared with model control (MC), **p < 0.01).

Flow cytometry was applied to detect the number of CD4+ T cells producing IFN-γ+ and IL-4+ (Figure 7). The proinflammatory factor, IFN-γ+ of CD4+ T cells in DSS-induced NASH rats increased significantly (*p* < 0.01), compared with the NC group. In comparison with MC group, the IFN-γ+ of the PC and the JZG groups substantially decreased (*p* < 0.01). Also, IL-4+ secreted by CD4+ T cells was significantly (*p* < 0.01) lowered in the MC group comparable to the NC. However, the PC and JZG groups increased substantially (*p* < 0.01), wherein it was more similar to the NC group.

### Expression of TLR-4 and MyD88 proteins

**Figure 8.**
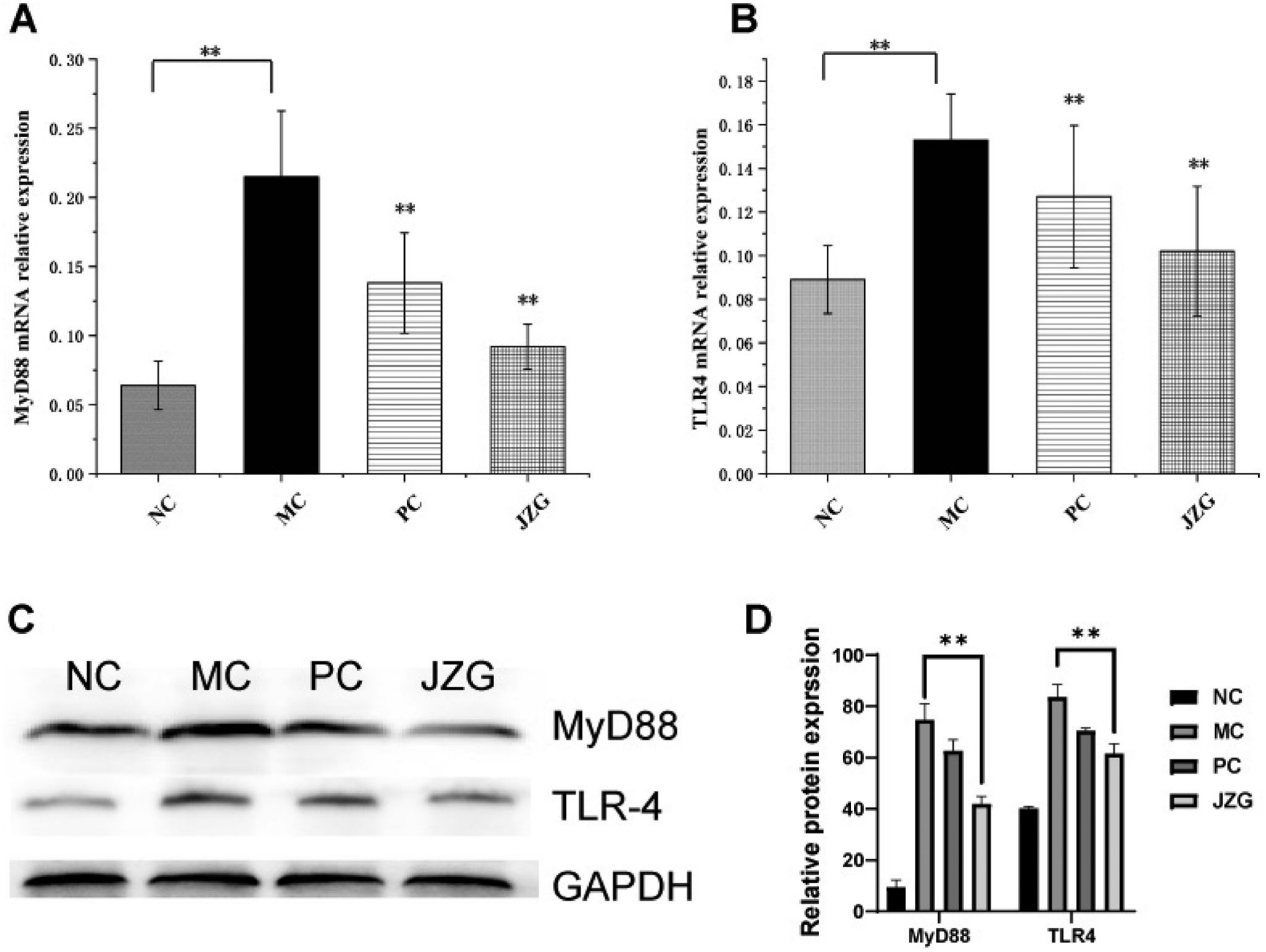
mRNA and protein expression of toll-like receptor 4 (TLR-4) and myeloid differentiation primary response 88 (MyD88) (compared with model control (MC), **p < 0.01).

As displayed in Figure 8, the detection of TLR-4 and MyD88 mRNA expressions was performed with quantitative real-time PCR and Western blot shown in Supplementary 2. Compared with the NC group, the relative expression of TLR-4 and MyD88 in MC increased significantly (*p* < 0.01). However, in comparison with MC, the relative expression of TLR-44 and MyD88 in PC and JZG groups substantially (*p* < 0.01) decreased. Meanwhile, the relative expression of TLR-4 and MyD88 in JZG group was lower than that of the PC group. Altogether, JZG supplementation may inhibit inflammatory response in rats with NASH.

## Discussion

Biochemically, NAFLD is a metabolic stress-linked liver injury, which is closely associated with IR and genetic susceptibility (Lo et al. 2015; Yu et al. 2015) amid being regarded as a common chronic liver disease worldwide. The pathogenesis of the disease has not yet been fully explained but it is generally accepted as the concept of ‘double strike’. First, it is postulated to be mainly caused by lipid deposition in hepatocytes with concomitant IR and lipid metabolism disorders as well as consequent increased susceptibility of the liver to inflammation and various injurious factors. However, oxidative stress and abnormal cytokines, which induce hepatic inflammation, hepatocyte degeneration and necrosis, hepatic fibrosis and even cirrhosis, underlie the second hypothesis. In the present study, NASH model was induced with DSS as stated elsewhere (Cheng et al. 2018). Usually, DSS-induced NASH is a manifestation of progressive liver injury, often characterized by steatosis of hepatocytes, intralobular inflammation and balloon hepatic degeneration with or without fibrosis (Carrier et al. 2016). The observation of liver enlargement and steatosis in the model group suggests that NASH model was successfully established.

Pathomechanistically, free radical damage is an important factor in the promotion of the occurrence and development of NASH (Ziamajidi et al. 2013; Ore and Akinloye 2019). Indeed, previous work has reported increased oxidative stress in NASH patients (Baskol et al. 2015). In this regard, antioxidant treatment coupled with increased total antioxidant status is considered as treatment options for NASH patients. Elevated serum ALT is a marker of inflammation and oxidative stress. Usually, ALT and AST are considered as the biomarker for injury to liver (Derakhshesh et al. 2019). The MDA is one of the degradative products of polyunsaturated fatty acid peroxide, which can cause cross-linking polymerization of nucleic acid, protein and other endogenous macromolecules, as well as induce cytotoxicity (Xie et al. 2017). Contrarily, SOD is an important antioxidant biomarker, which detoxifies the normal reactive oxygen species (Boland et al. 2018). Noticeably, treatment with JZG effectively reduced the plasma levels of ALT, AST, LDL-C, TC and TG as well as serum endotoxin in NASH rats, while the contents of SOD and HDL-C was increased (Figure 4). This phenomenon may lead to the promotion of free radical scavenging as well as reduction in the level of MDA and subsequent decrease in the direct damage of hepatocytes by MDA. Collectively, these results indicated that NASH rats suffered from impaired liver function. Consequently, histopathological examination showed that the JZG treatment remarkably reduced steatosis and inflammatory cell infiltration in liver tissue of model rats, as well as protected the hepatocytes. Actually, JZG has been studied on lipid-induced hepatitis in animal models. Wang et al. posited that JZG (828 mg/kg/d) showed anti-steatotic effect through the inhibition of liver X receptor-(LXR)-sterol regulatory element binding protein-1c (SREBP-1c) pathway (LXRα- SREBP-1c) activation. In their review, Zhang et al. (2020) summarized the potential of JZG to protect liver against NAFLD, especially the formula substantially reduced liver fat content, inflammation and oxidative stress. The aforementioned findings on JZG inspired the current work. Also, the potential of probiotics to treat hepatic steatosis has been explored in recent times. Several strains have been used in this regard, namely *Lactobacilli*, *Bifidobacteria*, *Streptococci*, *Enterococci*, *Lactococcus*, etc. In this work, the probiotic, *L. lactis* was chosen for this work because of the reported potential of this strain to attenuate metabolic-induced liver disorders and directly activate plasmacytoid DCs (Sugimura et al. 2013).

In normal liver, DCs were the immature type (Mbongue et al. 2017), while the expression efficiency of co-stimulatory molecules (CD80+, CD86+ and MHC-II+) in the immature DCs was very low. Based on the phenotypic values of CD80+ %, CD86+ % and MCH-II+ % in the MC group, the success of establishing NASH model in rats was demonstrated. In addition, DCs could secrete a variety of cytokines which participate in the regulation of immune function. Different DCs subgroups play varied regulatory roles in the differentiation and function of Th1 and Th2 (Bouteau et al. 2019), while Th cells regulate immunity through secretion of cytokines. Physiologically, cytokines produced during immune response are known to be involved in the formation of hepatic fibrosis, namely IFN-γ, IL-4 and IL-10 (Paulovicova et al. 2015). Also, IFN-γ and IL-4 are considered as the representative cytokines of Th1 and Th2 cells, respectively (Hwang 2017). Besides, IL-4+ secreted by DC could promote the differentiation of Th0 cells into Th2 cells and mediates immune response in fluids (Mastrangeli et al. 2009). Pathological examination of rats in the model group showed severe inflammatory exudation and infiltration of liver and colon, blockage of inflammatory embolus, abnormal increase in CD80/CD86 detection value, and significant difference in related cytokine detection value compared with the NC group. The above observation showed that the phenotypic expression of DCs was imbalance in NASH rats. This finding suggests that immune cells dysregulation may play central role in NASH pathogenesis. Besides, the exposure of liver to high amounts of bacterial products like endotoxin may activate the innate immune system which therefore initiates cascade of proinflammatory response that culminates in hepatic inflammation and fibrosis. Thus, drugs that target the immune cells of gut-liver axis may serve as an effective therapeutic option for NASH. Herein, a significant lowered content of IFN-γ was observed in JZG treated group compared with the MC group, while IL-4 level was substantially higher in JZG than that of the MC group. The expression of Th2 cytokines in the NASH model rats was too low, albeit that of the Th1 cells being higher. After supplementation with JZG, the DCs induced naive CD4+ T cells to differentiate into Th1 cells to protect intestinal mucosal immune barrier.

Actually, the activation of inflammation-related pathways is a major component in the progression of fatty liver fibrosis and cirrhosis. Importantly, the LPS induced TLR-4 activation is a central event in the pathology of alcoholic liver disease (ALD), and has many similar pathological features with NASH (Reichold et al. 2012; du Plessis et al. 2016). As a significant factor in innate immunity induction, TLR-4 is widely distributed in immune and non-immune cells. It has been reported (Hu et al. 2013; Nguyen et al. 2013) that TLR-4 induces the activation of NF-κB downstream of the pathway through Toll/IL-1 receptor domain (TIR domain) and MyD88 to produce inflammatory factors. Functionally, TLR-NF-κB signalling pathway mainly mediates the promotion of the synthesis of cytokines synthesis and NF-κB activation, which concomitantly increased the expression of TLR-4 in NASH rat model. Consequently, activated TLR-4 promoted the release of inflammatory factors through activation and regulation of NF-κB, thus inducing high inflammatory response. This phenomenon suggests that TLR-4 inflammatory pathway may participate in the development of NAFLD, while the activation of Kupffer cells by LPS via TLR4/MyD88 signalling pathway can trigger IR. Compared with the MC group, the expression of TLR-4 and MyD88 decreased significantly after JZG intervention. This suggests the possibility of the enteric mucosa DCs disrupting intestinal mucosal immune barrier in NASH rat model via TLR-4/MyD88 signalling pathway.

## Conclusions

In this study, a rat model of NASH was established by high fat diet DSS. Through observation via H&E staining of the model rat liver, the biochemical markers of the rats in each group were detected, which proved that the NASH model rat was successfully established. Administration of JZG reduced the level of biochemical markers (AST, ALT, MDA, LDL-C, TC and TG) in plasma of NASH rats, as well as decreased liver lipids, while liver function and pathological changes of liver tissue were remarkably improved. These results suggest that JZG had a certain therapeutic effect on experimental induced NAFLD. Obviously, JZG could reduce damage to immunologic barrier of intestinal mucosa in NASH rats with concomitant promotion of the maturation of intestinal mucosal DCs, and DCs-induced differentiation of naive CD4+ T cells into Th1 cells for the protection of intestinal mucosal immune barrier. More importantly, the mechanism underlying anti-NAFLD activity of JZG may be related to possible inhibition of TLR-4/MyD88 signalling pathway.
